# A Systematic Review of Childhood Maltreatment Assessments in Population-Representative Surveys Since 1990

**DOI:** 10.1371/journal.pone.0123366

**Published:** 2015-05-18

**Authors:** Wendy Hovdestad, Aimée Campeau, Dawn Potter, Lil Tonmyr

**Affiliations:** 1 Public Health Agency of Canada, Government of Canada, Ottawa, Canada; 2 Ottawa, Canada; Indiana University and Moi University, UNITED STATES

## Abstract

**Background:**

Population-representative surveys that assess childhood maltreatment and health are a valuable resource to explore the implications of child maltreatment for population health. Systematic identification and evaluation of such surveys is needed to facilitate optimal use of their data and to inform future research.

**Objectives:**

To inform researchers of the existence and nature of population-representative surveys relevant to understanding links between childhood maltreatment and health; to evaluate the assessment of childhood maltreatment in this body of work.

**Methods:**

We included surveys that: 1) were representative of the non-institutionalized population of any size nation or of any geopolitical region ≥ 10 million people; 2) included a broad age range (≥ 40 years); 3) measured health; 4) assessed childhood maltreatment retrospectively; and 5) were conducted since 1990. We used Internet and database searching (including CINAHL, Embase, ERIC, Global Health, MEDLINE, PsycINFO, Scopus, Social Policy and Practice: January 1990 to March 2014), expert consultation, and other means to identify surveys and associated documentation. Translations of non-English survey content were verified by fluent readers of survey languages. We developed checklists to abstract and evaluate childhood maltreatment content.

**Results:**

Fifty-four surveys from 39 countries met inclusion criteria. Sample sizes ranged from 1,287-51,945 and response rates from 15%-96%. Thirteen surveys assessed neglect, 15 emotional abuse; 18 exposure to family violence; 26 physical abuse; 48 sexual abuse. Fourteen surveys assessed more than three types; six of these were conducted since 2010. In nine surveys childhood maltreatment assessments were detailed (+10 items for at least one type of maltreatment). Seven surveys’ assessments had known reliability and/or validity.

**Conclusions and Implications:**

Data from 54 surveys can be used to explore the population health relevance of child maltreatment. Assessment of childhood maltreatment is not comprehensive but there is evidence of recent improvement.

## Introduction

Child maltreatment has been defined by the Public Health Agency of Canada as neglect, exposure to intimate partner violence, emotional maltreatment, physical and sexual abuse [1]. The American Centers for Disease Control similarly defines child maltreatment as abuse or neglect of someone under age 18 years by any person in a custodial role [2]. Child maltreatment is recognized as a serious problem around the world. Cumulative childhood prevalence estimates range from 1% (intrusive sexual abuse of boys) to 35% (assaults including serious threats), depending on the maltreatment type, respondents’ gender, the geographical region, and other factors [3]. These may be underestimates, given that some interviewees with previously documented maltreatment provide false negatives [4–7]. Adults with histories of childhood maltreatment are more likely than those without such histories to experience a variety of negative health outcomes, and these associations persist when effects due to variables such as income are statistically controlled [8–10]. A wide variety of population-representative surveys are relevant to health, whether they focus on health behaviours (e.g., alcohol use, sexual behaviour), on specific health outcomes, or on social issues with health implications (e.g., criminal victimization). If such surveys include childhood maltreatment measures, they can illuminate the long-term population health implications of the maltreatment of children.

Although causality cannot be inferred from cross-sectional surveys, it has been recently argued that retrospective population-representative community based surveys have an important role to play in understanding child maltreatment [11]. Such surveys also allow the study of health-relevant outcomes that may be undocumented (e.g., re-victimization, untreated illness), in administrative medical and social services databases. In addition, they allow the exploration of research questions that are potentially difficult to address with child samples due to ethical and reporting requirements [12,13]. Nonetheless, population-representative surveys are usually limited to the non-institutionalized population with a fixed household address and may be limited to fluent speakers of the dominant regional language. These exclusions may cause underestimation of the strength of the associations between adversity-related predictor and health outcome variables [14,15]. Although concerns have been raised about the use of retrospective reports of childhood maltreatment [5], arguments by Kendell-Tackett and Becker-Blease [16] and analyses of data from New Zealand, American, British, and German samples are reassuring [9,17–19]. Recent work has found, for example, that psychological adjustment is related to adverse childhood experiences in the same way whether adversity is assessed prospectively or retrospectively [19].

Some issues with using population representative surveys as a means to understand child maltreatment and population health are specific to the surveys’ childhood maltreatment assessments. For example, the definition of a type of maltreatment may be left to the respondents (e.g., “Were you ever physically abused?”) rather than being defined by specific behaviours. Items to assess maltreatment may be of unknown reliability and validity. Some types of child maltreatment (e.g., neglect, emotional abuse) are studied less often than others [3] and surveys’ inclusion of multiple types of childhood maltreatment is also an important issue.

Spurred in part by earlier reports of issues encountered in efforts to include childhood maltreatment and other highly personal questions in health surveys [20–22] and following from earlier reviews [23,24], this is a systematic review and evaluation of the childhood maltreatment assessments in population-representative surveys with any health content, worldwide, since 1990. The first objective is to provide a resource that informs health researchers of the existence and nature of surveys that assessed childhood maltreatment in order to facilitate secondary data analyses. The second objective is to describe and evaluate childhood maltreatment assessments that have been used on earlier population-representative surveys. This paper is intended as a resource to facilitate planning of future surveys in Canada and worldwide. To the best of our knowledge no similar resource exists.

## Methods

This systematic review was done according to PRISMA guidelines (see S1 PRISMA Checklist).

### Search Strategy

**Fig 1:**
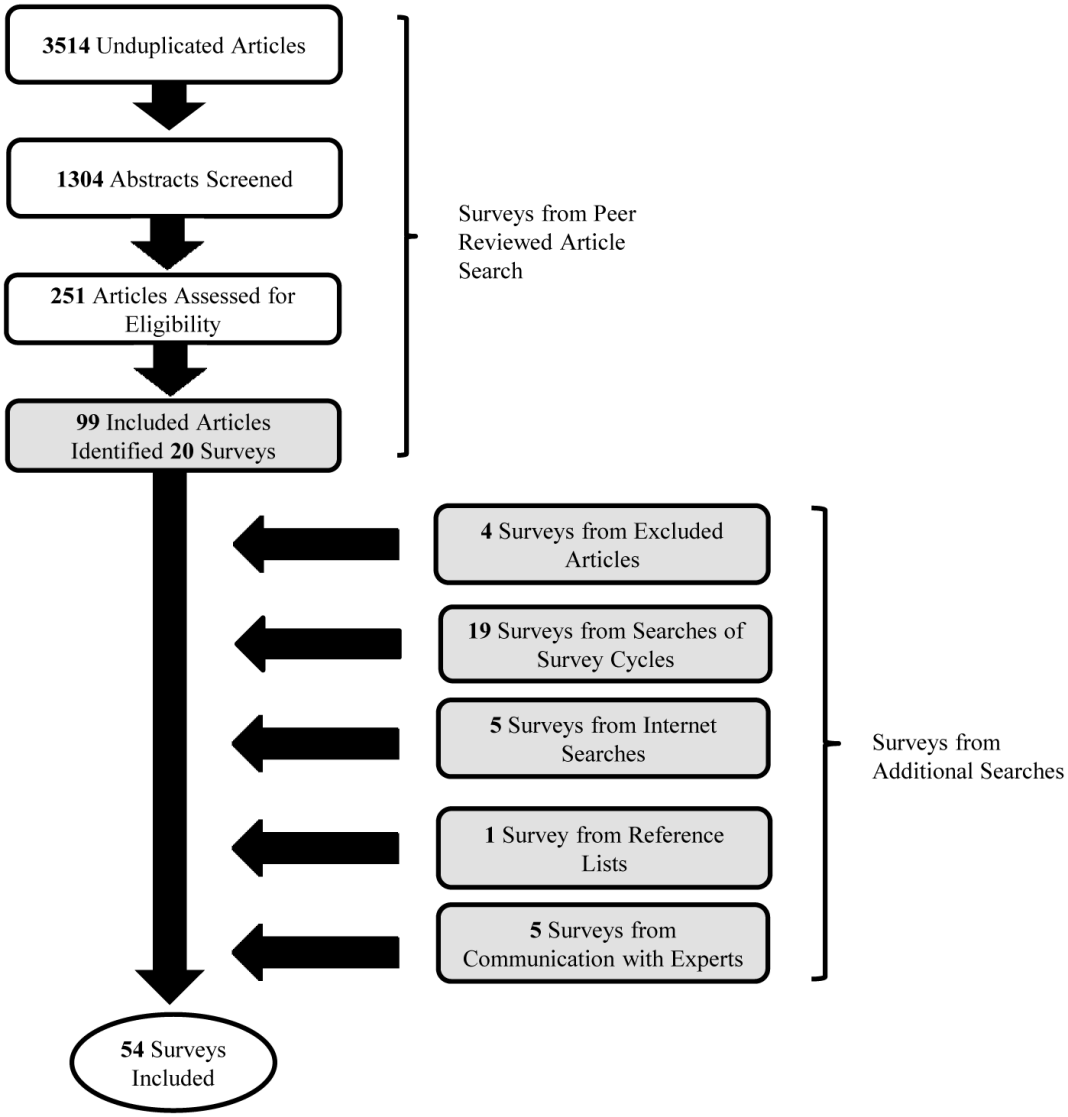
Survey Identification and Inclusion Process. Unshaded boxes represent database searches of peer-reviewed articles. Shaded boxes represent sources of included surveys.

Fig 1 shows our means of including surveys. We began by searching citation databases to identify peer-reviewed articles that used data from relevant surveys (January 1990-March 2014). Cinahl, Embase, ERIC, Global Health, MEDLINE, PsycINFO, Scopus, Social Policy and Practice were searched with assistance from a librarian.

A sample search string was: ((TITLE-ABS-KEY-AUTH((survey* W/3 health) OR (representati* W/3 survey*) OR (household* W/3 survey*) OR (general* W/3 survey*) OR (morbid* W/3 survey*))) AND TITLE-ABS-KEY((child* AND victimization) OR (child* W/3 abuse*) OR (child* W/3 neglect*) OR (child* W/5 assault*) OR (child* W/2 violence) OR (child W/3 maltreat*) OR (child* AND punishment*) OR (pumitiv* W/3 experienc*) OR (child* AND witness* AND violen*))) OR (((TITLE-ABS-KEY-AUTH((survey* W/3 health) OR (representati* W/3 survey*) OR (household* W/3 survey*) OR (general* W/3 survey*) OR (morbid* W/3 survey*)))) AND (TITLE-ABS-KEY(national* AND child*)) AND (TITLE-ABS-KEY(victimizat* OR victimisat* OR (child* W/3 abus*) OR (child* W/3 maltreat*) OR (child* W/3 neglect*) OR (domest* W/3 violenc*) OR (domest* W/3 violent*) OR (famil* W/3 violen*) OR (partner* W/3 violen*) OR (sex* W/3 assault*) OR (sex* W/3 abus*) OR (sex* W/3 maltreat*) OR (psycholog* W/3 assault*) OR (psycholog* W/3 abus*) OR (psycholog* W/3 maltreat*) OR (emotion* W/3 assault*) OR (emotion* W/3 abus*) OR (emotion* W/3 maltreat*) OR ((witness* OR expos* OR exposed*) AND violen*) OR "physical punishment" OR punitive OR "physical abuse"))).

In the initial search, two teams of two reviewers independently screened titles and abstracts. After this training phase, titles and abstracts were screened by one reviewer. Inclusion criteria for articles identified in this way were as stated below. Five additional steps were taken to identify relevant surveys not described in the 99 included articles. Excluded articles were re-checked as articles pertaining to subpopulations did, at times, utilize representative surveys. Internet searches were performed using titles of all included surveys to ensure additional cycles that met criteria were not overlooked. Internet searches were conducted using an abbreviated list of search strings to identify potential new surveys. A list of included surveys was shared with two electronic discussion forums populated by child maltreatment experts. Communication with experts led to the identification of additional relevant surveys. Finally, we searched reference lists from included and excluded articles, along with references from research bibliographies on survey websites.

Additional materials pertaining to reliability and validity of surveys’ childhood maltreatment assessments were obtained by other Internet searching (using key words from each survey title in combination with “reliability” and “validity”).

### Survey Selection

Population representative surveys including assessment of both childhood maltreatment and health were eligible for inclusion. We defined population representative surveys as those which were described that way by users of the data (i.e., authors of articles), which had been sampled and weighted in order to accurately reflect the members of the entire population.

We defined childhood maltreatment as respondents’ experiences before age 18 years involving family or caregiver-related emotional or physical neglect, emotional or physical abuse. The initial review protocol specified assessments specific to childhood exposure to intimate partner violence but was adapted to include violence within the family in which victims and/or perpetrators were unspecified (See S1 Protocol). We included any sexual abuse before age 18, not exclusively acts committed by a caregiver. Our choices here with regard to how to define childhood maltreatment reflect our child welfare and public health informed understandings that child sexual abuse (where a child is a person under age 18 years) is different from other forms of child maltreatment. Sexual assault of children, especially girls, is common, and has important health impacts whether the perpetrators are intra- or extra-familial. Some forms of child maltreatment (e.g., neglect, emotional abuse) can only occur within ongoing relationships. Sexual abuse is different, in that strangers and acquaintances can and do victimize children this way.

No minimum quality criteria were applied to childhood maltreatment assessments; items posed to survey respondents only needed to correspond to any one of the five maltreatment subtypes [1].

Health was defined broadly, including mental and physical health, self-esteem, health care utilization, alcohol or substance use, injury, and re-victimization (but not violence perpetration nor experience of criminal sanctions). Fourteen non-English surveys were included because the survey was described in an English article (see search strategy) or because communication with survey administrators and/or article authors was possible. Surveys were included if they were representative of the household population of a sovereign nation or if they were representative of a distinct geopolitical region of at least 10 million people. Due to our broad public health focus and the need to limit the scope of the review, surveys were excluded if respondent ages spanned less than 40 years (e.g., adolescents and young adults only) or if respondents were representative only of a subpopulation (e.g., women).

#### Data Collection Process

The complete instruments were obtained for review for all but seven of the included surveys. For the seven surveys, experts confirmed that questions pertaining to childhood maltreatment were repeated verbatim from earlier cycles for which the complete instruments had been obtained, or provided excerpts containing the childhood maltreatment content. Survey methods, geographical coverage information, maltreatment type, and characteristics of the maltreatment assessment measures (e.g., reliability, validity) were extracted from research and methods articles, survey websites (where available), survey instruments, and from personal communications with survey administrators and data users. Survey instruments were searched for additional child maltreatment content not described in associated articles. Five checklists were developed and adapted as necessary to reflect concepts used on surveys to assess childhood maltreatment. The key shared domains of our five checklists were item count, self-defined maltreatment, behaviours constituting maltreatment, and indicators of severity. The checklists correspond exactly to the column headings, used below. Our use of checklists to describe and evaluate surveys’ childhood maltreatment assessment is in keeping with earlier recommendations that the best tools are simple checklists, specific to the content, with a small number of key domains [25]. Better quality of childhood maltreatment assessment was indicated by use of multiple rather than single items, behaviourally-specific rather than self-defined items, use of items with known reliability and validity, and assessment of multiple rather than single types of childhood maltreatment. Survey instruments and associated documentation were reviewed independently by at least two authors to ensure that all relevant content was accurately extracted. At all stages of data extraction, disagreements were rare and were resolved by discussion to consensus.

## Results

Table 1 summarizes the 54 included surveys, conducted in 39 countries. The table presents the surveys in chronological order, with multi-cycle surveys grouped together. The majority of surveys were conducted in high income countries (e.g., the United States, Canada, several European countries), followed by middle to low-middle income countries (e.g., Brazil, China), and a low income country (i.e., Uganda). The Ugandan survey was conducted as part of the Gender, Alcohol and Culture International Study (GENACIS). More surveys were conducted in the United States than any other country (43%). The sample sizes ranged from 1,287–51,945 and the response rates from 15%-96%. Sexual abuse was assessed most often, followed by physical abuse, exposure to family violence, emotional abuse, and neglect. Four American surveys included all five types of childhood maltreatment: the National Comorbidity Survey (NCS), the Behavioral Risk Factors Surveillance System (BRFSS) California 2008, 2009, and the second cycle of the National Epidemiologic Survey on Alcohol and Related Conditions (NESARC2).

**Table 1:** Fifty-Four Representative Population Health, Social and Victimization Surveys since 1990 Included in this Review, in Chronological Order.

Survey	Country	Year(s) conducted	Mode of Administration	*N*	Response Rate^a^	Age	Childhood maltreatment type				
							NG	EA	EFV	PA	SA
The Ontario Health Survey Mental Health Supplement (OHSUP) [26,27]	Canada	1990–91	PAPI	9 953	67%	15+	•	–	–	•	•
National Comorbidity Survey (NCS) [28,29]	United States	1990–92	PAPI	8 098	82%	15–54	•	•	•	•	•
*NCS-2*[28,30]		*2001–03*	*CAPI*	*5 001*	*88%*	*26–67*	•	–	–	–	–
National Health and Social Life Survey (NHSLS) [21,31]	United States	1992	PI, PAPQ	3 159	79%	18–59	–	–	–	–	•
General Social Survey (GSS) [32–37]	Canada	1999	CATI^b^	25 876	81%	15+	–	–	–	–	•
		2009	CATI	19 422	62%	15+	–	–	–	•	•
		2014	CATI, CAPI	-^c^	-	15+	–	–	•	•	•
National Survey of Midlife Development (MIDUS) [38,39]	United States	1995–96	CATI, PAPQ	3 034	61%	25–74	•	•	–	•	–
*MIDUS-2*[40,41]		*2004–06*	*CATI*, *PAPQ*	*1 805*	*57%*	*32–85*	–	–	–	–	•
National Violence Against Women Survey (NVAWS) [42,43]	United States	1995–96	CATI	16 005	70%	18+	–	–	–	•	•
National Alcohol Survey (NAS) [44–51]	United States	1995	PI	4 803	77%	18+	–	–	•	•	–
		2000	CATI	7 612	58%	18+	–	–	–	–	•
		2005	CATI	6 919	56%	18+	–	–	–	•	•
		2010	CATI	7 969	52%	18+	–	–	–	•	•
[Sex in Sweden] (SIS) [52,53]	Sweden	1996	PAPQ	2 810	59%	18–74	–	–	–	–	•
[The Netherlands Mental Health & Incidence Study] (NEMESIS) [54–57]	The Netherlands	1996	PAPI	7 076	70%	18–64	•	•	–	•	•
		2007–09	PI	6 646	65%	18–64	•	•	–	•	•
[Chinese Health and Family Life Survey] (CHFLS) [58,59]	China^d^	1999–00	CASI	3 821	76%	20–64	–	–	–	–	•
National Study of Health, Intimacy, and Social Relations (NSHIS) [60,61]	Australia	2000	CATI	1 784	58%	18–59	–	–	–	–	•
Gender, Alcohol and Culture: An International Study (GENACIS) [62,63]	multinational^e^	2000–07	interview^f^	44 219	15%-96%^g^	varied^h^	–	–	–	–	•
[Health Barometer] (Barometer) [64–69]	France	2000	CATI	13 685	93%	12–75	–	–	–	–	•
		2005	CATI	30 514	63%	12–75	–	–	–	–	•
		2010	CATI	27 653	-^i^	15–85	–	–	–	–	•
[Health and Morbidity Survey] (SUSY) [70]	Denmark	2000	PAPQ	14 278	64%	16+	–	–	–	–	•
Sexual Abuse and Violence in Ireland (SAVI) [71,72]	Ireland	2001	PATI	3 120	71%	18–90	–	–	–	–	•
Australian Study of Health and Relationships (ASHR) [73–75]	Australia	2001–02	CATI	19 307	73%	16–59	–	–	–	–	•
		2011–12	CATI	20 000	-^j^	16–69	–	–	–	–	•
Second Injury Control and Risk Survey (ICARIS-2) [76]	United States	2001–03	TI	9 684	48%	18+	–	–	•	•	•
World Mental Health Surveys (WMHS) [77,78]	Multinational^k^	2001–09	CAPI, PAPI	51 945	45%-88%	18+^l^	•	–	•	•	•
Behavioral Risk Factor Surveillance System (BRFSS)	United States										
Texas [79,80]		2002	CATI	6 262	46%	18+	–	•	•	•	•
Ohio [81,82]		2003	CATI	3 875	41%	18+	–	–	–	–	•
Florida [82,83]		2008	CATI	11 069	50%	18+	–	–	•	–	–
California [83,84]		2008	CATI	12 047	38%	18+	•	•	•	•	•
California [84,85]		2009	CATI	18 120	43%	18+	•	•	•	•	•
California [84,86]		2010	CATI	18 587	43%	18+	–	–	–	–	•
Florida [82,86]		2010	CATI	37 419	44%	18+	–	–	•	–	–
Pennsylvania [86,87]		2010	CATI	11 822	47%	18+	–	•	•	•	•
California [84,88]		2011	CATI	18 892^m^	35%	18+	–	•	•	•	•
National Epidemiologic Survey on Alcohol and Related Conditions—Wave 2 (NESARC2) [89,90]	United States	2004–05	PAPI	34 653	87%	20+	•	•	•	•	•
{*German Surveys*} (USUMA) [91–97]	Germany	2005	PAPQ^n^	2 341	65%^o^	18–93	–	–	–	–	•
		2006	PI	1 287	62%	14+	•	•	–	•	–
		2007	PAPQ	2 510	62%	14–93	–	–	–	–	•
		2008	PAPQ	5 033	62%	14+	–	–	–	–	•
[Brazilian National Alcohol Survey] (BNAS) [98–101]		2010	PI	2 504	56%	14+	•	•	–	•	•
	Brazil	2005–06	PAPI	3 007	66%	14+	–	–	•	•	•
		2011–12	PI	4 607	77%	14+	–	•	•	•	•
[Relationships and Sexuality] (RS) [102–104]	Netherlands	2005–06	Online	4 170	28%	19–70	–	–	–	–	•
		2008–09	Online	6 428	21%	15–70	–	–	–	–	•
Adult Psychiatric Morbidity Survey (APMS) [105,106]	United Kingdom	2007	CAPI, CASI	7 353	57%	16+	–	–	•	•	•
National Intimate Partner and Sexual Violence Survey (NISVS) [107–110]	United States	2010	CATI	16 507	~31% ^p^	18+	–	–	–	–	•
		2011	CATI	12 727	33%	18+	–	–	–	–	•
Canadian Community Health Survey (CCHS) Mental Health [111,112]	Canada	2012	CAPI, CATI	25 113	69%	15+	–	–	•	–	•
Korean General Social Survey (KGSS) [113, 114]	Korea	2012	PI	1 396	56%	18+	•	•	–	•	•
{English Survey} (UK) [115]	United Kingdom	2013	CAPI,PAPQ	4 010	54%	18–69	–	•	•	•	•
					% of total		24	28	34	48	89

Ns represent unique samples except for NCS-2 (2001–03) and MIDUS-2 (2004–06) which were follow-up surveys, *italicized*. NG = Neglect, EA = Emotional abuse, EFV = Exposure to family violence, PA = Physical abuse, SA = Sexual abuse, PAPI = Paper and pencil interview, PAPQ = Paper and pencil self-completed questionnaire, CAPI = Computer assisted personal interview, CATI = Computer assisted telephone interview, CASI = Computer assisted self-interview, PATI = Paper and pencil telephone interview, PI = Personal interview of undetermined type, TI = Telephone interview of undetermined type.

^a^ Unless otherwise noted, response rate is percent of eligible respondents who completed the survey. The conditional RR is presented for the NCS-2 (only those who were in NCS & have not since died are possible respondents.)

^b^ CAPI in some remote regions.

^c^ At the time of this review, data collection was still ongoing, therefore, N and response rate are estimated.

^d^ The CHFLS was administered in 18 provinces: Liaoning, Heilongjiang, Jilin, Gansu, Inner Mongolia, Shaanxi, Hebei, Beijing, Tianjin, Henan, Shandong, Hunan, Anhui, Jiangsu, Zhejiang, Shanghai, Fujian, and Guangdong (http://popcenter.uchicago.edu/data/chfls.shtml).

^e^ The 18 countries in which the GENACIS was administered with the SA questions were: Argentina, Belize, Brazil, Canada, Costa Rica, Czech Republic, India, Kazakhstan, New Zealand, Nicaragua, Nigeria, Peru, Spain, Sri Lanka, Uganda, United Kingdom (including the Isle of Man), United States of America, Uruguay.

^f^ One country used a postal survey, all others used interview. Surveys were administered in person except the US (28% telephone) and Australia and Canada (100% telephone).

^g^ Response rate was not available for 7/19 countries.

^h^ Usually 18+.

^i^ Refusal rate reported as 40%.

^j^ Response rate not yet known.

^k^ Based on included articles, childhood maltreatment questions are known to have been included in the WMHS in these 22 countries: Belgium, Brazil[Sao Paulo], Bulgaria, China, Columbia, France, Germany, India, Israel, Italy, Japan, Lebanon, Mexico, Netherlands, New Zealand, Nigeria, Romania, South Africa, Spain, Turkey, Ukraine, United States, although all countries did not include all types of childhood maltreatment.

^l^ Age of youngest participants varied between countries, ranging from 16–21 years but was typically either 18 or 21.

^m^ Calculation based on landline and cell phone distributions (Table 5a and 5b) in Summary Data Quality Report 2011.

^n^ USUMA 2005, 2007, 2008 data collection took place in the context of a personal household visit.

^o^ Beutel et al. report this as the response rate but also describe 65% as the percentage of the sample that could be contacted, which may not be the same as the percentage who completed an interview.

^p^ The overall weighted response rate ranged from 27.5% to 33.6%.

As shown in Tables 2–6, for all types of childhood maltreatment some surveys included items relying on respondents’ self-definition (e.g., “As a child, did you ever witness parent/guardian abuse by their spouse/partner?”). Self-definition was most common with sexual abuse (48%), and nine of the 48 surveys assessing sexual abuse included exclusively a self-definition item. Single-items were used to assess exposure to family violence and emotional abuse over 50% of the time. Detailed assessments of childhood maltreatment (+10 items) were usually of sexual abuse (23% of surveys that included sexual abuse) with one of these (National Violence Against Women Survey) also assessing physical abuse in detail. NESARC2 assessed neglect with more than 10 items and also assessed the other four types of maltreatment with six or more items. The majority of the surveys included assessment of various aspects of maltreatment severity such as frequency, immediate harm, perpetrator identity, and age at occurrence.

**Table 2:** Description and Assessment of Neglect Measures on 13 Included Surveys, in Chronological Order.

Survey/year/# items	Self-defined	Behaviours included in neglect items															Additional severityinformation			
		Unsupervised too young	Effort into watching over (R)	Failed to protect from known danger	Made to do age inappropriate chores	Parents too drunk/ high to take care of the family	Hungry/ meals not prepared	Go w/out things you needed (clothing/ shoes/hygiene)	Ignore medical needs, fail to give treatment	Not listened to/ problems ignored/ lack of attention/ support	Family looked out for each other (R)	Given/Felt love & affection (R)	Family a source of strength (R)	Close knit family (R)	Close, Confiding relationship w parents/ other adult (R)	Family helped me feel important/ special, believed in me, wanted success for me (R)	Frequency	Immediate Harm	Perpetrator identity	Specification of childhood
OHSUP/1990–91/1 [27]	–	–	–	–	–	–	–	–	–	–	–	–	–	–	•	–	–	–	-	C
NCS/1990–92/5 [29]	•	–	•	–	–	–	–	–	–	–	–	–	–	–	•	–	–	–	-	C
NCS2/2001/5 [30]	–	•	–	–	•	–	•	•	•	–	–	–	–	–	–	–	•	–	P	C
MIDUS/1995–96/4 [39]	–	–	•	–	–	–	–	–	–	•^a^	–	•	–	–	•^b^	–	•	–	-	C
NEMESIS/1996/2 [55]	–	–	–	–	–	–	–	–	–	•	–	–	–	–	–	–	•	–	ID	<16
NEMESIS-2/2007–09/3 [57]	–	–	–	–	–	–	–	–	–	•	–	–	–	–	–	–	•	–	ID	<16
WMHS/2001–09/6–10 [78]	–	•	•	–	•	–	•	•	•	–	–	•	–	–	•	–	•	–	P	C
NESARC2/2004–05/11–15 [90]	•	•	–	–	•	–	•	•	•	–	–	–	•	•	–	•	•	–	P, U	10^c^, <18, RD
USUMA/2006/1 [94]	–	–	–	–	–	–	–	–	–	•^d^	–	–	–	–	–	–	•	–	P	-^e^
USUMA/2010/6–10 [97]	–	–	–	•^f^	–	•	•	•	•^g^	–	•	•	•	•	–	•	•	–	U	<18
BRFSS California/2008/1 [84]	–	–	–	•	–	–	•	•	•	–	–	–	–	–	–	–	–	–	P	C, <18
BRFSS California/2009/1 [84]	–	–	–	•	–	–	•	•	•	–	–	–	–	–	–	–	–	–	P	C, <18
KGGS/2012/5 [114]	–	–	–	–	–	–	–	–	–	•	–	•	–	–	–	–	–	•	ID	<18

There are two indications in a cell when some items on the survey are assessed in one way and some in another (e.g., C, <18). • = the survey assessed this characteristic on one or more items; – = the survey did not assess this characteristic on any item; (R) = reverse coded item; P = perpetrator is defined within one or more items as parent or person who raised the respondent; ID = respondents identified the perpetrator in terms of their relationship; U = perpetrator is unspecified (e.g., “people in my family”); C = when respondent was growing up, during childhood or adolescence; RD = precise age at occurrence of maltreating experience was recorded and thus researchers can define; <# = survey defines childhood as the years before this birthday.

^a^ Reverse scored: Item read: “How much time and attention did she/he give you when you needed it?”

^b^ Item read: “How much could you confide in mother/father about things that were bothering you?"

^c^ Age 10 years was specified for “unsupervised too young.”

^d^ Item read: “You were comforted by your parents when you were sad?”

^e^ Unable to identify specification of childhood.

^f^ Reverse scored. Item read “I knew that there was someone to take care of me and protect me.”

^g^ Reverse scored. Item read “There was someone to take me to the doctor if I needed it.”

**Table 3:** Description and Assessment of Emotional Abuse Measures on 15 Included Surveys, in Chronological Order.

Survey/year/# items	Self-defined	Behaviours included in emotional abuse items													Additional severity information			
		Favoured siblings	Stomped out of room	Sulked or refused to talk to you	Insulted	Swore at, cursed	Did or said something to spite or hurt feelings	Punished you unjustly	Threatened to throw something at you	Threatened to hit or harm you, other, or possession	Blackmailed you	Acted such that you feared physical injury	Acted out (e.g., kicking, smashing of things)	Other	Frequency	Immediate Harm	Perpetrator identity	Specification of childhood
NCS/1990–92/2 [29]	–	–	•	•	•	•	•	–	–	•	-	–	•	–	•	–	ID	C
MIDUS I/1995–96/1 [39]	–	–	•	•	•	•	•	–	–	•	–	–	•	–	•	–	ID	C
NEMESIS/1996/2 [55]	–	•	–	–	–	•	–	•	–	–	•	–	–	–	•	–	ID	<16
NEMESIS-2/2007–09/3 [57]	–	•	–	–	•	–	–	•	–	•	•	–	–	•^a^	•	–	ID	<16
BRFSS Texas/2002/2 [80]	–	–	–	–	•	•	•	–	–	–	–	•	–	–	–	–	P, A	<18
BRFSS California/2009/1 [84]	–	–	–	–	•	•	•	–	–	–	–	–	–	–	–	–	P, A	C
BRFSS California/2008/1 [84]	–	–	–	–	•	•	•	–	–	–	–	–	–	–	–	–	P, A	C
BRFSS California/2011/1 [84]	–	–	–	–	•	•	•	–	–	–	–	–	–	–	•	–	P, A	C, <18
BRFSS Pennsylvania/2010/1 [87]	–	–	–	–	•	•	•	–	–	–	–	–	–	–	•	–	P	<18
NESARC2/2004–05/3 [90]	–	–	–	–	•	•	•	–	•	•	–	•	–	–	•	–	P	<18
USUMA/2006/1 [94]	–	–	–	–	–	–	–	•	–	–	–	–	–	–	•	–	P	C
USUMA/2010/5 [97]	•	–	–	–	•	–	•	–	–	–	–	–	–	•^b^	•	•	P	<18
BNADS/2011–12/1 [101]	–	–	–	–	•	–	•^c^	–	–	–	–	–	–	–	•	–	P	C, <18
KGGS/2012/4 [114]	–	–	–	–	•	–	–	–	–	–	–	–	–	•^d^	–	•	ID	<18
UK/2013/1 [115]	–	–	–	–	•	•	•	–	–	–	–	–	–	–	•	–	P, A	<18

There are two indications in a cell when some items on the survey are assessed in one way and some in another (e.g., P,A). • = the survey assessed this characteristic on one or more items; – = the survey did not assess this characteristic on any item; P = perpetrator is defined within one or more items as parent or person who raised the respondent; A = survey specifies that an adult in the respondent’s home was the perpetrator; ID = respondents identified the perpetrator in terms of their relationship; C = when respondent was growing up, during childhood or adolescence; <# = survey defines childhood as the years before this birthday.

^a^ Item included “abuse” and “belittle.”

^b^ Item read: “I felt that someone in family hated me”; “You thought that your parents wished you had never been born.”

^c^ Item read: “…humiliated you publically.”

^d^ Item read: “I was often scorned or got contempt.”

**Table 4:** Description and Assessment of Measures Assessing Exposure to Family Violence on 18 Included Surveys, in Chronological Order.

Survey/year/*#* items		Behaviours included in exposure to family violence items																Additional Severity Information		
	Self-defined	Directly witnessed	Insulted/ cursed	Did/ said something to spite	Angry withdrawal	Acted out (kick, smash things)	Threat harm/ threat physical attack	Threw something at someone	Pushed/ grabbed/ shoved	Slapped/ hit	Kicked/ bitten	Punched/ hit with a fist	Choked/ attempted drowning	Hit with object	Beat up	Burned/scalded	Weapon used	Frequency	Victim/ perpetrator identity	Specification of childhood
NCS/1990–92/3 [29]	–	–	•	•	•	•	•	•	•	•	•	•	•	•	•	•	–	•	IPV	C
NAS/1995/4 [45]	–	•	–	–	–	–	•	–	–	–	–	–	–	–	–	–	–	•	IPV	C
ICARIS2/2001–03/1 [76]	•	•	–	–	–	–	–	–	–	–	–	–	–	–	–	–	–	–	IPV	C
WMHS/2001–09/2 [78]	–	•	–	–	–	–	–	•	•	•	–	–	–	–	–	–	–	•	U	C, RD
BRFSS Texas/2002/4 [80]	–	–	–	–	–	–	•	•	•	•	•	•	–	•	•	–	•	•	M	<18
BRFSS Florida/2008/1 [82]	–	–	–	–	–	–	–	•	•	•	–	–	–	–	–	–	–	–	IPV, A	<18
BRFSS Florida/2010/1 [82]	–	–	–	–	–	–	–	•	•	•	–	–	–	–	–	–	–	–	IPV, A	<18
BRFSS California/2008/1 [84]	–	–	–	–	–	–	–	–	–	•	–	•	–	–	•	–	–	–	IPV, A	C, <18
BRFSS California/2009/1 [84]	–	–	–	–	–	–	–	–	–	•	–	•	–	–	•	–	–	–	IPV, A	C, <18
BRFSS California/2011/1 [84]	–	–	–	–	–	–	–	–	–	•	•	•	–	–	•	–	–	•	IPV, A	C, <18
BRFSS Pennsylvania/2010/1 [87]	–	–	–	–	–	–	–	–	–	•	•	•	–	–	•	–	–	•	IPV, A	<18
NESARC2/2004–05/6–10 [90]	–	•	–	–	–	–	•	•	•	•	•	•	–	•	•^a^	–	•	•	U, M	<18, RD
BNAS/2005–06/2 [99]	•	•	–	–	–	–	•	–	–	–	–	–	–	–	–	–	–	•	IPV^b^	C
BNDAS/2011–12/2 [101]	•	•	–	–	–	–	•	–	–	–	–	–	–	–	–	–	–	•	IPV^c^	<18, C
APMS/2007/2 [106]	•	–	–	–	–	–	–	–	–	–	–	–	–	–	–	–	–	–	U	<16
CCHS-MH/2012/1 [112]	–	•	–	–	–	–	–	–	–	•	–	–	–	–	–	–	–	•	IPV, A	<16
UK/2013/1 [115]	–	–	–	–	–	–	–	–	–	•	•	•	–	–	•	–	–	•	IPV, A	<18
GSS/2014/1 [37]	–	•	–	–	–	–	–	–	–	•	–	–	–	–	–	–	–	•	IPV, A	<15

There are two indications in a cell when some items on the survey are assessed in one way and some in another (e.g., C, RD). • = the survey assessed this characteristic on one or more items; – = the survey did not assess this characteristic on any item; U = perpetrator and victim are unspecified (e.g., “violence in the home”); IPV = violence was between respondent’s parents or respondent’s parent and the parent’s intimate partner; M = violence against respondent’s mother (or other female caretaker); A = survey specifies that “an adult” was the perpetrator or victim; C = when respondent was growing up, during childhood or adolescence; RD = precise age at occurrence of maltreating experience was recorded and thus researchers can define; <# = survey defines childhood as the years before this birthday.

^a^ Item read: “repeatedly hit.”

^b^ Item read: “physically assault each other or other person.”

^c^ Item read: “physically assault each other or other person.”

**Table 5:** Description and Assessment of Physical Abuse Measures on 26 Included Surveys, in Chronological Order.

Survey/year/# items	Self-defined	Behaviours included in physical abuse items														Additional severity information			
		Threw something at	Pushed, grabbed, shoved	Slapped, hit	Kicked, bit	Choked, attempted drown	Hit w/ object	Beat up	Threatened w/ gun or weapon	Gun / weapon used	Burned, scalded	Punched, hit w/ fist	Spanked, physically punished	Hair pulled	Other	Frequency	Immediate harm	Perpetrator identity	Specification of childhood
OHSUP/1990–91/6–10 [27]	–	•	•	•	•	•	•	–	–	–	•	•	•	–	•	•	•	ID	C
NCS/1990–92/6–10 [29]	•	•	•	•	•	•	•	•	–	–	•	•	•	–	–	•	–	ID	RD
MIDUS I/1995–96/2 [39]	–	•	•	•	•	•	•	•	–	–	•	•	–	–	–	•	–	ID	C
NVAWS/1995–96/16+ [43]	–	•	•	•	•	•	•	•	•	•	–	–	–	•	–	–	•	ID	C, RD^a^
NAS/1995/4 [45]	–	–	–	–	–	–	•	•	•	•	•	–	–	–	–	–	–	P	C
NAS/2005/4 [49]	–	–	–	–	–	–	•	•	•	•	•	–	–	–	–	–	•	ID	C, <18, RD
NAS/2010/6–10 [51]	–	–	–	–	–	–	•	•	•	•	•	–	–	–	–	–	•	ID	C, <18, RD
NEMESIS/1996/2 [55]	–	–	–	•	•	–	•	–	–	–	–	–	–	–	•	•	–	ID	<16
NEMESIS-2/2007–09/3 [57]	–	–	–	–	•	–	•	•	–	–	•	–	X	–	–	•	–	ID	<16
ICARIS 2/2001–03/1 [76]	–	–	•	•	–	–	–	–	–	–	–	–	•	–	–	–	•	P, A	C
WMHS/2001–09/6–10 [78]	–	•	•	•	•	•	–	•	•	–	•	•	•	–	–	•	–	ID	C, RD
BRFSS Texas/2002/2 [80]	–	–	•	•	–	–	–	–	–	–	–	–	–	–	–	•	•	P	<18
BRFSS California/2008/1 [84]	•	–	–	•	•	–	–	•	–	–	–	–	X	–	–	–	–	P, A	C, <18
BRFSS California/2009/1 [84]	•	–	–	•	•	–	–	•	–	–	–	–	X	–	–	–	–	P, A	C, <18
BRFSS California/2011/1 [84]	•	–	–	•	•	–	–	•	–	–	–	–	X	–	–	•	–	P, A	C, <18
BRFSS Pennsylvania/2010/1 [87]	•	–	–	•	•	–	–	•	–	–	–	–	–	–	–	•	–	P	<18
NESARC2/2004–05/6–10 [90]	•	–	•	•	–	–	–	•	–	–	–	–	–	–	–	•	•	P	<18, RD
BNAS/2005–06/6–10 [99]	–	–	–	•	–	–	•	–	•	•	•	–	–	–	–	–	•	P	C
BNDAS/2011–12/6–10 [101]	–	–	•	–	–	–	•	•	•	•	•	–	–	–	•^b^	•	–	P	C, <18
USUMA/2006/1 [94]	–	–	–	•	–	–	–	–	–	–	–	–	–	–	–	•	–	P	C
USUMA/2010/5 [97]	•	–	–	–	–	–	•	•	–	–	–	–	•	–	–	•	•	–	<18
APMS/2007/2 [106]	–	–	–	–	–	–	–	•	–	–	–	–	–	–	–	–	–	P	<16, RD
GSS/2009/4 [35]	•^c^	–	–	–	–	–	–	–	–	–	–	–	–	–	–	–	–	ID	<15
GSS/2014/4 [37]	•	•	•	•	•	•	•	–	–	–	•	•	X	–	–	•	–	ID, A	<15
KGSS/2012/7 [114]	–	•	•	•	•	–	–	–	–	–	•	•	•	–	–	–	•	ID	<18
UK/2013/1 [115]	•	–	–	•	•	–	–	•	–	–	–	–	X	–	–	•	–	P, A	<18

There are multiple indications in a cell when some items on the survey are assessed in one way and some in another (e.g., C, RD). • = the survey assessed this characteristic on one or more items; – = the survey did not assess this characteristic on any item; P = perpetrator is defined within one or more items as parent or person who raised the respondent; A = survey specifies that an adult in the respondent’s home was the perpetrator; ID = respondents identified the perpetrator in terms of their relationship; C = when respondent was growing up, during childhood or adolescence; RD = precise age at occurrence of maltreating experience was recorded and thus researchers can define; <# = survey defines childhood as the years before this birthday; X = an item explicitly ruled out spanking.

^a^ Respondents indicated their age at the last time a victimization experience happened to them.

^b^ Item read: “…scratched, pinched, knocked.”

^c^ item read: “assault (face-to-face threat or assault with or without a weapon but neither theft nor attempted theft of property).”

**Table 6:** Description and Assessment of Sexual Abuse Measures on 48 Included Surveys, in Chronological Order.

Survey/year/# items	Self-defined	Behaviours included in sexual abuse items													Additional severity information			
		Noncontact	Threat to rape you	Kiss sexually	Photo, video taken	Molestation/ touch sex parts of your body	Made to touch other	Made to masturbate	Rub genitals against you	Receive oral/ genital contact	Attempt sex assault (oral/ anal/ vaginal)	Rape (oral/ anal/ vaginal penetration)	Insert object	Other	Frequency	Immediate Harm	Perpetrator identity	Specification of childhood
OHSUP/1990–1/6–10 [27]	•	•	•	–	–	•	–	–	–	–	•	–	–	–	–	•	ID	C
NCS/1990–92/6–10 [29]	–	–	–	–	–	•	–	–	–	–	–	•	–	–	•	–	ID	RD
NHSLS/1992/16+ [31]	•	–	–	•	–	•	•	–	•	•	–	•	–	•^a^	•	–	ID	RD
GSS/1999/2 [33]	–	–	–	–	–	•	–	–	–	–	•	•	–	–	•	–	–	RD
GSS/2009/4 [35]	–	–	–	–	–	•	–	–	–	–	•	•	–	–	–	–	ID	<15
GSS/2014/3 [37]	•	–	–	•	–	•	–	–	–	–	•	–	–	–	•	–	ID, A	<15
NVAWS/1995–96/11–15 [43]	–	–	–	–	–	–	–	–	–	•	•	•	•	–	–	•	ID	RD
SIS/1996/16+ [53]	–	•	–	–	•	•	•	•	–	•	–	•	•	–	•	–	ID	RD
NEMESIS/1996/3 [55]	–	–	–	–	–	•	•	–	–	–	–	–	–	–	•	–	ID	<16
NEMESIS-2/2007–09/11–15 [57]	•	•	–	–	–	•	–	•	–	–	•	•	–	–	•	–	ID	<16
CHFLS/1999–00/16+ [59]	–	–	–	–	–	•	–	–	–	–	–	•	–	–	•^b^	•	ID	<14, RD
NSHIS/2000/11–15 [61]	–	•	–	–	–	•	•	–	•	•	•	•	–	•^c^	•	–	–	<16, RD
NAS/2000/2 [47]	–	–	–	–	–	•	•	–	–	–	–	•	–	–	–	–	–	C,<18, RD
NAS/2005/4 [49]	–	–	–	–	–	•	•	–	–	–	–	•	–	–	–	•	ID	C, <18, RD
NAS/2010/6–10 [51]	–	–	–	–	–	•	•	–	–	–	–	•	–	–	–	•	ID	C, <18, RD
GENACIS/2000/2 [63]	•	•	–	–	–	–	–	–	–	–	–	–	–	–	•	–	ID^d^	<16
Barometer/2000/4 [65]	•	–	–	–	–	•	–	–	–	–	•	•	–	–	•	–	–	RD
Barometer/2005/4 [67]	–	–	–	–	–	•	–	–	–	–	•	•	–	–	•	–	–	RD
Barometer/2010/2 [69]	–	–	–	–	–	–	–	–	–	–	–	•	–	–	–	–	–	RD
SUSY/2000/2 [70]	•	–	–	–	–	–	–	–	–	–	–	–	–	–	–	–	ID	<18^e^
ASHR/2001/3 [74]	•	–	–	–	–	–	–	–	–	–	–	–	–	–	•	–	–	RD
ASHR/2011–12/3 [75]	•	–	–	–	–	–	–	–	–	–	–	–	–	–	•	–	–	RD
SAVI/2001/16+ [72]	–	•	–	–	•	•	•	–	•	–	•	•	•	–	•	•	ID	<17
ICARIS-2/2001–03/11–15 [76]	–	–	–	–	–	–	–	–	–	–	–	•	–	–	–	–	ID	RD
WMHS/2001–09/6–10 [78]	–	–	–	–	–	•	–	–	–	–	–	•	•	–	•	–	ID	RD
BRFSS Texas/2002/4 [80]	–	–	–	–	–	•	•	–	–	–	•	•	–	–	–	–	A	<18
BRFSS Ohio/2003/2 [82]	•	–	–	–	–	–	–	–	–	–	•	•	–	–	–	–	ID	<18
BRFSS California/2008/1 [84]	–	–	–	–	–	•	•	–	–	–	–	•	–	–	–	–	A	C
BRFSS California/2009/1 [84]	–	–	–	–	–	•	•	–	–	–	–	•	–	–	–	–	A	C
BRFSS California/2010/1 [84]	•	–	–	–	–	–	–	–	–	–	–	–	–	–	–	–	–	<18
BRFSS California/2011/4 [84]	•	–	–	–	–	•	•	–	–	–	–	•	–	–	•	–	A	C, <18
BRFSS Pennsylvania/2010/3 [87]	–	–	–	–	–	•	•	–	–	–	–	•	–	–	•	–	A	<18
MIDUS II/2004–5/3 [41]	•	–	–	–	–	–	–	–	–	–	–	•	–	–	•	•	–	RD
NESARC II/2004–05/6–10 [90]	–	–	–	–	–	•	•	–	–	–	•	•	–	–	•	–	–	RD, <18
USUMA/2005/1 [92]	•	–	–	–	–	–	–	–	–	–	–	–	–	–	–	–	–	<14
USUMA/2007/1 [92]	•	–	–	–	–	–	–	–	–	–	–	–	–	–	–	–	–	<14
USUMA/2008/1 [92]	•	–	–	–	–	–	–	–	–	–	–	–	–	–	–	–	–	<14
USUMA/2010/5 [97]	•	•	–	–	–	•	–	–	–	–	•	–	–	–	–	–	–	<18
BNAS/2005–06/1 [99]	•	–	–	–	–	–	–	–	–	–	–	–	–	–	–	–	P	C
BNDAS/2011–12/3 [101]	–	–	–	–	–	•	–	–	–	–	–	•	–	•^f^	–	–	ID	C, <18
RS/2005–06/2^g^ [102]	•	•	–	–	–	•	–	–	–	–	–	–	–	–	–	–	–	<16
RS/2008–09/16+ [104]	•	•	–	–	–	•	•	•	–	•	•	•	–	–	•	–	ID	<16, RD
APMS/2007/5 [106]	•	•	–	–	–	•	•	–	–	–	–	•	–	–	–	–	–	<16, RD
NISVS/2010/16+ [108]	–	•	•	•	•	•	–	–	–	•	•	•	•	–	•	•	ID	RD
NISVS/2011/16+ [110]	–	•	–	•	•	•	–	–	–	•	•	•	•	–	•	•	ID	RD
CCHS-MH/2012/2 [112]	•	–	–	•	–	•	–	–	–	–	•	–	–	–	•	•	A	<16
KGSS/2012/3 [114]	•	–	–	•	–	•	–	–	–	–	–	•	–	–	–	•	ID	<18
UK/2013/3 [115]	–	–	–	–	–	•	•	–	–	–	–	•	–	–	•	–	A	<18

There are two indications in a cell when some items on the survey are assessed in one way and some in another (e.g., C, RD). • = the survey assessed this characteristic on one or more items; – = the survey did not assess this characteristic on any item; P = perpetrator is defined within one or more items as parent or person who raised the respondent; ID = respondents identified the perpetrator in terms of their relationship; A = survey specifies that “an adult” or someone 5+ years than respondent was the perpetrator; C = when respondent was growing up, during childhood or adolescence; RD = precise age at occurrence of maltreating experience was recorded and thus researchers can define; <# = survey defines childhood as the years before this birthday.

^a^ Item read: “Active anal intercourse, rubbing genitals on perpetrator’s body…”

^b^ Age at onset and offset were recorded.

^c^ Item read: “Before the age of 16, did someone try to sexually arouse you when you did not want them to?”

^d^ Family vs. non-family perpetrators were distinguished.

^e^ Childhood defined as under the age of 13. Adolescence defined 13–17.

^f^ Item read: “Have you ever received money for sex before the age of 18?”

^g^ Estimated, 2 known items.

Table 2 describes 13 surveys that assessed neglect. Items assessing respondents’ experiences of going without food and other necessities and of having unmet medical needs were most common, followed by items about emotional neglect such as lack of attention and absence of close relationships with caregivers.

Table 3 describes 15 surveys that assessed respondents’ experiences of emotional abuse. Most surveys assessed verbally abusive behaviours such as insults, swearing at, cursing at or doing or saying something to spite or hurt feelings.

Table 4 describes 18 surveys that assessed exposure to family violence. Common behaviours included exposure to a family member being slapped or hit, being pushed, shoved or grabbed, or having something thrown at them. Six surveys dealt with aspects of being exposed to the emotional abuse of another family member in the form of threats of harm.

Table 5 describes 26 surveys that assessed physical abuse. The behaviours assessed most often were: slapped or hit, beaten up, hit with an object, and burned or scalded.

Table 6 describes 48 surveys that assessed sexual abuse. The behaviours assessed most often were rape (oral and/or anal and/or vaginal penetration) followed by molestation.

Included articles contained information about the reliability, validity, or lack thereof, of the childhood maltreatment assessments used in the NCS, the Ontario Health Survey—Mental Health Supplement (OHSUP), NESARC2 and the Korean GSS. Searches for additional materials uncovered evidence for reliability and validity of the childhood maltreatment assessment used on the 2010 survey conducted by the German statistical company, *Unabhangiger Service für Umfragen*, *Methoden und Analysen* (USUMA 2010), which used a German version of the well-validated Childhood Trauma Questionnaire [116]. In addition, the exposure to family violence item on the Canadian Community Health Survey—Mental Health (CCHS 2012) was drawn from a measure with established reliability and validity [117,118], as were the physical abuse and exposure to family violence items on the General Social Survey—2014 [117, 118]. Many surveys used items modified from existing measures (e.g., the Conflict Tactics Scale [119,120]).

## Discussion

The objectives of this review were to provide information about the existence and nature of population health surveys that assess childhood maltreatment and to provide an evaluation of those assessments.

Our review allows discussion of some general characteristics of the included surveys. We identified more surveys in later years compared to earlier years that assessed childhood maltreatment and this may reflect either an increasing number of population-representative surveys or an increasing proportion of such surveys that included childhood maltreatment. Given the successful uses of several high profile surveys to explore the long-term health importance of child maltreatment [121,122] and statements about child maltreatment prevention as preventive of chronic disease and other costly health issues [123,124], there may be a new willingness among sponsors of recent surveys to include childhood maltreatment assessment.

In the early 1990s, interview method was predominantly personal, in contrast to surveys conducted since 2009, which were almost all computer-assisted telephone interviews. The only online (web based) surveys that met inclusion criteria were conducted recently (2005–2009). Given evidence that research participants may prefer to disclose victimization using a computer rather than to an interviewer [125], online surveys may have potential to advance research in this area. However, based on sampling theory and a simulation study, Bethlehem concluded that self-selection web surveys have “no role” in creation of accurate estimates of population characteristics [126]. Thus, the utility of online surveys for future research in this area is uncertain.

Childhood maltreatment was conceptualized using exclusively self-defined items on eight surveys that assessed sexual abuse, two for physical abuse, and two for exposure to family violence; self-defined items were also sometimes used in concert with behaviour-based items. Enhancing quality by use of items that are behaviour specific (versus those that are self-defined or interpretative) has been previously discussed [117,127].

Despite longstanding calls for use of high quality measures [24], we found evidence for the reliability and/or validity of the childhood maltreatment assessments on only seven of the 54 included surveys. The Composite International Diagnostic Inventory (CIDI) formed a part of several surveys, and includes a checklist of life events that includes childhood maltreatment. The CIDI has well established validity and reliability [128], but Kessler et al [129] have noted the potential difficulties in assessing life adversities with checklists. Further examination of the specific items used in the surveys indicated that few were used in formats identical to those tested for reliability and/or validity; the psychometric properties of nearly all measures were uncertain.

Maltreatment types commonly co-occur, and study of single types in isolation has been decried [130–132]. Assessment of multiple types of maltreatment is important for understanding which forms of maltreatment co-occur and how different forms of maltreatment, and their co-occurrence, are risk factors for later health outcomes [130]. Although 14 surveys included more than three forms of childhood maltreatment, we found that half the surveys assessed a single type of childhood maltreatment, and almost always that single type was sexual abuse. We confirmed earlier findings [133,134] that sexual abuse is researched more than other types of maltreatment. Note, however, that our definition of sexual abuse was broader than that for other types of maltreatment. Also, the preponderance of sexual abuse assessments on surveys may be explained, in part, by research related to sexual health and HIV transmission risk factors [21].

Limitations of our work include the following: The total number of surveys identified was not recorded. In keeping with earlier recommendations [25], our assessment checklist tools were carefully developed, but their reliability and validity have not been tested. Stoltenborgh et al.’s review [135] noted the inherent conceptual difficulties of the definition and measurement of child neglect; our neglect tool content should in particular be validated by further research.

Although we searched comprehensively, it is unlikely that all existing surveys have been identified, especially non-English ones. We limited our scope by excluding surveys and articles in which the sample was subnational or in which a segment of the population with an age range smaller than 40 years was targeted because they did not meet our definition of representativeness [136,137].

Despite our international perspective, our inclusion criteria may have resulted in selection of material constructed around concerns held predominantly by Western scientists and policy makers. For example, surveys concerned with child morbidity and mortality were excluded [138–140], although such work may be of key concern in low income countries. Items on the neglect checklist may be particularly Western-centric, in that omissions of care and nurturing seen as “neglect” in one culture may not be seen as problematic in another [141]. Tausig [142] discussed the importance of methodological factors and cultural contexts in understanding health estimates derived from international surveys, and a similar perspective may be useful in this context considering that most survey assessments of childhood maltreatment originate from one culture.

A final limitation of this review is that some information of potential interest was not coded, such as accessibility of the data for secondary analyses, which cannot be assumed to be straightforward, as noted for example by Thompson and Xiajie [143]. We did not assess the availability of measures of non-maltreatment childhood adversity (e.g., poverty) nor childhood supportive relationships (except where such measures, reverse coded, could be seen as indicators of emotional neglect) despite evidence that both are important to adult health outcomes [144–146].

This systematic review, enhanced by Internet searches and consultations with experts, represents a unique assessment of 54 diverse population-representative surveys conducted internationally since 1990 that measured both childhood maltreatment and adult health. This study has a number of strengths. Due to a weighty reliance on grey literature, publication bias is probably not an issue. The process we followed enhanced the quality of the review; basing our tools on survey content, as it emerged, allowed for the diversity of content to be represented; coders represented multiple disciplines; disagreements were resolved by consensus. In addition, in terms of content extraction each identified survey was thoroughly searched; our tools captured diverse surveys’ highly varied childhood maltreatment content from within multiple modules (e.g., “post-traumatic stress disorder”, “childhood”, “background information”, “sexual violence”, “life event history”).

The absence of validated measures and failure to assess multiple types of childhood maltreatment are two concerns in this body of work. Important questions for future work are: Can both these concerns be addressed within surveys that must have the minimum possible administrative and response burdens? If not, which concern is more important to address? Evidence indicates that single-item measures of childhood maltreatment are associated with under-reporting. However, other research indicates no clear effect of maltreatment means of assessment on the strength of the relation with health outcomes [147] and single-item measures of childhood maltreatment predict adult health outcomes [137].

Routine inclusion of childhood maltreatment assessment on surveys with any health content would allow further understanding of child maltreatment as a risk factor for various adverse health outcomes throughout the lifespan including those with high social costs, such as chronic disease. Our review demonstrates the feasibility of inclusion and provides diverse examples of previous assessments. A future paper will examine the strength of the observed relationships between childhood maltreatment and health outcome measures (e.g., mental illness, chronic illness such as cancer).

Population-representative surveys are a key source of data to inform public policy about child maltreatment as a preventable problem associated with negative health outcomes. To our knowledge this is a unique, comprehensive search and description of health and social surveys and assessment of their childhood maltreatment content. It is our hope that health researchers who recognize the importance of childhood maltreatment will benefit from knowing on what surveys childhood maltreatment items have been included, and the nature of the surveys and of the items.

## Supporting Information

S1 PRISMA Checklist PRISMA 2009 Checklist. (DOC)

S1 Protocol Research Protocol for A Systematic Review of Childhood Maltreatment Assessments in Population-Representative Surveys Since 1990. (DOCX)
