# Supplementary material for: A Systematic Review of Childhood Maltreatment Assessments in Population-Representative Surveys Since 1990
Source: PLoS One. 2015 May 18;10(5):e0123366. doi: 10.1371/journal.pone.0123366 (PMC4436275; doi:10.1371/journal.pone.0123366)
Supplement: S1 Protocol — (DOCX) [file pone.0123366.s002.docx]

Research Protocol for A Systematic Review of Childhood Maltreatment Assessments in Population-Representative Surveys Since 1990

Table of Contents:

1. Statement of Purpose
2. Selection Criteria
3. Search Strategies
   1. Article search
   2. Data extraction
   3. Supplementary Searches for Surveys
      1. To locate survey instruments
      2. Search within surveys to identify childhood maltreatment content
4. Survey Coding Definitions & Rules
   1. Table 1 (survey information)
   2. Tables 2-6 (common childhood maltreatment content)
   3. Table 2: Neglect
   4. Table 3: Emotional Abuse
   5. Table 4: Exposure to Family Violence
   6. Table 5: Physical Abuse
   7. Table 6: Sexual Abuse
5. Reliability/validity
   1. Supplemental search
6. Protocol References

1. Statement of Purpose

This systematic review will identify surveys conducted since 1990 that have at least one item assessing childhood maltreatment and at least one item assessing health (broadly defined). Its purposes are: 1) to inform health researchers of the existence and nature of population representative surveys relevant to understanding links between childhood maltreatment and health; 2) to evaluate the assessment of childhood maltreatment in this body of work, in terms of quality of measures, range of types of childhood maltreatment assessed, etc.

This protocol outlines the steps, strategies, and decision rules that were utilized to conduct the review.

2. Selection Criteria

Articles and surveys were assessed using the same inclusion/exclusion criteria^[[1]](#footnote-1)^. Peer reviewed research articles were included if they used the complete sample of a survey that met inclusion criteria (e.g., not restricted to one gender, not restricted to an age subsample). Articles were excluded if they combined childhood maltreatment items with items assessing other adverse childhood experiences (e.g., poverty) such that effects due to childhood maltreatment could not be disentangled. Meeting summaries, reports, and dissertations/theses were excluded from the review. Surveys were excluded if respondent ages spanned less than 40 years (e.g., young adults ages 18-35) or if respondents were representative only of a subpopulation (e.g., women).

Eligible for inclusion:

- Population-representative surveys including assessment of both childhood maltreatment and health.
- Surveys representative of the household population of any size sovereign nation.
- Surveys representative of the household population of a distinct geopolitical region of at least 10 million people (e.g., the province of Ontario, Canada; the state of Texas, United States of America).
- We defined childhood maltreatment as respondents’ experiences before age 18 years involving any non-consensual sexual contact and/or interaction and/or any sexual assault, and/or family or caregiver-related emotional and/or physical neglect, emotional and/or physical abuse, and/or exposure to family violence.
  - The initial review protocol specified items that assessed childhood exposure to intimate partner violence but this was adapted to include violence within the family in which victims and/or perpetrators were unspecified.
  - For details about how the five types of maltreatment were defined, see below. (Survey Coding Definitions & Rules, Tables 2-6)
- No minimum quality criteria were applied to childhood maltreatment assessments; items posed to survey respondents only needed to correspond to any one of the five maltreatment subtypes. See “Survey Coding Definitions” below for more information.
- Health was defined broadly, including mental and physical health, self-esteem, health care utilization, alcohol or substance use, injury, and re-victimization (but not violence perpetration nor experience of criminal sanctions).
- Fourteen non-English surveys were included because the survey was described in an English article or communication with survey administrators and/or article authors was possible.

3. Search Strategies

To identify surveys, two strategies were used: article search and survey search. The article search identified research articles from abstracts databases. The surveys that were sources of the data analysed in those articles were then included in this review.

a. Article Searches:

Database(s): Econlit 1961 to September 2012, Embase 1974 to 2012 October 09, Global Health 1973 to September 2012, Ovid MEDLINE(R), Ovid MEDLINE(R) In-Process & Other Non-Indexed Citations, Ovid MEDLINE(R) Daily and Ovid OLDMEDLINE(R) 1946 to Present, PsycINFO 1987 to October Week 1 2012, Social Policy and Practice 201207

The article search was updated in March 2014.
Key Search Terms

| **#** | **Searches** |  |
| --- | --- | --- |
| 1 | (child$ and (victimizat* or victimisat*)).mp. |  |
| 2 | (child$ adj4 (abuse$ or maltreat$ or violen$ or neglect$ or assault$)).ti,ab. |  |
| 3 | (child$ and (physical$ adj2 punishment$)).mp. |  |
| 4 | (child$ and punitiv$ and experienc$).mp. |  |
| 5 | (survey$ adj3 (health or representativ$ or household$ or house?hold$ or general$ or morbid$)).mp. |  |
| 6 | or/1-4 |  |
| 7 | 5 and 6 |  |
| 8 | (national$ and child$).mp. |  |
| 9 | (victimizat$ or victimisat$ or (child$ adj2 (abus$ or maltreat$ or neglect$)) or ((domest$ or famil$ or partner$) and (violenc$ or violent$)) or ((sex$ or psycholog$ or emotion$) adj2 (assault$ or abus$ or maltreat$)) or ((witness$ or expos$ or exposed$) and violen$) or ("physical punishment" or punitive or "physical abuse")).mp. |  |
| 10 | 5 and 8 and 9 |  |
| 11 | 7 or 10 |  |
| 12 | limit 11 to yr=2011-2012 |  |
| 13 | remove duplicates from 12 |  |

((TITLE-ABS-KEY-AUTH((survey* W/3 health) OR (representati* W/3 survey*) OR (household* W/3 survey*) OR (general* W/3 survey*) OR (morbid* W/3 survey*))) AND TITLE-ABS-KEY((child* AND victimization) OR (child* W/3 abuse*) OR (child* W/3 neglect*) OR (child* W/5 assault*) OR (child* W/2 violence) OR (child W/3 maltreat*) OR (child* AND punishment*) OR (pumitiv* W/3 experienc*) OR (child* AND witness* AND violen*))) OR (((TITLE-ABS-KEY-AUTH((survey* W/3 health) OR (representati* W/3 survey*) OR (household* W/3 survey*) OR (general* W/3 survey*) OR (morbid* W/3 survey*)))) AND (TITLE-ABS-KEY(national* AND child*)) AND (TITLE-ABS-KEY(victimizat* OR victimisat* OR (child* W/3 abus*) OR (child* W/3 maltreat*) OR (child* W/3 neglect*) OR (domest* W/3 violenc*) OR (domest* W/3 violent*) OR (famil* W/3 violen*) OR (partner* W/3 violen*) OR (sex* W/3 assault*) OR (sex* W/3 abus*) OR (sex* W/3 maltreat*) OR (psycholog* W/3 assault*) OR (psycholog* W/3 abus*) OR (psycholog* W/3 maltreat*) OR (emotion* W/3 assault*) OR (emotion* W/3 abus*) OR (emotion* W/3 maltreat*) OR ((witness* OR expos* OR exposed*) AND violen*) OR "physical punishment" OR punitive OR "physical abuse"))) AND (LIMIT-TO(PUBYEAR, 2012) OR LIMIT-TO(PUBYEAR, 2011))

b. Data extraction from articles:

The original abstract database search was conducted early in 2011, updated in September 2011, October 2012, and March 2014.

Initial training for article screening and data coding:

The titles and abstracts for 519 citations identified in the initial search were used to develop and train coders on screening criteria and coding. Senior authors were Wendy Hovdestad and Lil Tonmyr. Two teams of a senior author and a research assistant each reviewed half of the citations. One team (LT & AC) excluded 188 records and the other (WH & DP) excluded 205, to arrive at a pool of 126 articles for initial review. Erring on the side of inclusion in initial review, citations missing abstracts were obtained for further review, as were citations whose title or abstract did not indicate that the sample was not nationally representative. Fifty articles were coded by the teams, after which the research assistants read the other team’s coded articles and generated independent codes. Inter-rater reliability was excellent.

After initial training was complete, for all subsequent abstract database searches, one author prescreened all citations and excluded those that were irrelevant, those in which a special population (e.g., women, adolescents) was specified in the title, and those in which the focus was: child protection; child maltreatment and child health/mortality; child health or development in general; peer bullying; or a position paper or commentary rather than a data-based paper. Information about childhood maltreatment items, their psychometric properties, and survey characteristics was extracted from the articles, with consultations with a senior author as needed. For quality control, two readers independently coded three variables for each article, one of which coded the presence or absence in the article of information about the psychometric properties (reliability, validity) of the CM items. Inter-rater agreement was excellent. Disagreements were resolved by discussion.

Included articles often did not provide enough detail to permit evaluation of childhood maltreatment measures and thorough description of the surveys. Therefore internet searches for questionnaire content and contact with authors of included articles and survey administrators were undertaken. During these internet searches other nationally-representative surveys, not identified in the article search, were found to meet inclusion criteria. Also, some articles used data from surveys that met inclusion criteria but the articles themselves did not meet inclusion criteria (e.g., due to analysis of a survey’s subsample). To allow the scope of the review to match the original intent, the decision was thus made to report results of this review based on surveys, not articles. This decision made it necessary to supplement the abstracts database search with direct searches for surveys that were not described in included articles. (A future review of included articles, with additional data extraction pertaining to the findings, will summarize and assess what is known about childhood maltreatment and population health.)

c. Supplementary Searches for Surveys:

In addition to the article search, included surveys were identified in these ways:

- Excluded article search: articles excluded for reasons other than unrepresentativeness of the survey sample were searched for details about the survey (e.g., name, year conducted). If the available information about the survey indicated that the survey met inclusion criteria, the survey instrument was obtained (see “To locate survey instruments” below).
- Searches of survey cycles: if one cycle of a multi-cycle survey was included, an Internet search was undertaken to determine if other cycles had been conducted since 1990. If there was indication that childhood maltreatment content may have been included (e.g., cycle was titled “victimization,” “family”), the survey instrument was obtained (see “To locate survey instruments” below). If no such information about cycle themes was available, a sample of available cycles was reviewed.
- Internet searches: survey was identified searching using search terms abbreviated from citation search (e.g., “national” or “representative” and “child abuse” [and variants]), or a link to a survey was found unintentionally (e.g., while researching other cycles of a known survey). If there was indication that childhood maltreatment content may have been included (e.g., cycle was titled “victimization”), the survey instrument was obtained (see “To locate survey instruments” below).
- Reference list: included articles referred to a survey in introduction or discussion. If the available information about the survey indicated that the survey met inclusion criteria, the survey instrument was obtained (see “To locate survey instruments” below).
- Communication with experts: survey experts were contacted as necessary when necessary information could not be found in available articles or internet web sites. In several cases survey contacts provided the sections of the survey with childhood maltreatment content or confirmed that content from previous cycles was included in the new cycle. In addition, although polls of experts were conducted by messages sent to two electronic discussion forums populated by experts active in the field of child maltreatment, this did not result in identification of any additional surveys.
- The one exception to this protocol was the BRFSS. For these surveys, the state-added questions database was searched for all states with a population over 10 million (2013-2014).

Surveys identified in any of these five ways were deemed to have the potential to have items assessing any of the five types of CM content and were thus further examined according to “search within surveys,” below.

An additional search was conducted in October/November 2014 to identify any potential new cycles of all included surveys with less than a ten year pattern of reoccurrence. First, known survey websites were visited for updated content (e.g. reports about new cycles). Next, survey titles and years (e.g. 2013, 2014) were searched using a popular internet search engine. If no information was presented about a new cycle within the first 30 hits, the search for that survey was considered completed. If information about a new cycle was presented through this method, only those surveys completed before our project was expected to be published were pursued (e.g. National Alcohol Survey was not further pursued because it is in the field 2014-2015). If surveys were completed in time, our next efforts focused on obtaining the questionnaire. If questionnaires could not be obtained online, experts, website coordinators, or authors of studies based on these surveys were contacted.

3. c. i. To locate survey instruments for review and assessment:

- Follow up from reference list in included or excluded articles. If this was unsuccessful, then:
  - Internet searching with survey title (or, if title was unknown, search with “child abuse” (and variants), with country name, and date conducted) to locate url. Websites hosted by sponsoring organizations were also searched (e.g., in cases of multiple cycles). If this was unsuccessful, then:
  - Contact with authors of articles using the survey to learn url or to obtain a scanned or pdf copy of the survey instrument.

This three step process was successful for all but seven included surveys. For the seven surveys, experts (e.g., authors of articles using the data) confirmed that questions pertaining to child maltreatment were repeated verbatim from earlier cycles for which the complete instruments had been obtained, or provided excerpts of the survey instrument that contained the childhood maltreatment content.

No surveys known to meet inclusion criteria (e.g., based on published articles or reports that presented childhood maltreatment information) had to be excluded due to an inability to obtain the survey instrument.

3.c.ii. Search within surveys to identify childhood maltreatment content:

Some survey instruments were more than 50 pages long and some spanned multiple pdf or html files. Based on careful reading of several multipart survey instruments, 11 terms were identified from a larger set of possibilities as having utility in identifying childhood maltreatment content. To identify all childhood maltreatment content (not exclusively that noted in articles or reports using the survey data) we searched electronic copies of surveys with these terms: Assault, Child, Father, Growing, Hit, Molest, Mother, Parent, Rape, Sex, Spank. Survey instruments that were not electronically searchable were read in their entirety.

Exception: It was not possible to search foreign language surveys for each type of childhood maltreatment; only those types and assessments of those types that were described in an English-language article could be included.

Training for data extraction from surveys proceeded in a manner similar to that for articles, with two independent readers and multiple four-way discussions for content coding of the initial 30-40% of survey instruments. All survey instruments and associated documentation were reviewed independently by at least two authors to ensure that all relevant content was accurately abstracted. At all stages of data abstraction disagreements were rare and were resolved by discussion to consensus.

4. Survey Coding Definitions & Rules

4. a. Table 1 (Survey Information)

Information about years in which surveys were conducted, mode of administration, number and age range of participants, and response rate were extracted from methods articles wherever possible. If methods articles were unavailable, survey information was extracted from survey websites, included research articles, and other research articles using data from the pertinent survey. In cases of conflicting information, definitive sources (in order of highest authority first) were: methods articles, survey websites, and the most recent research article. For survey series in which a central body collected and stored information about each cycle, we used the source provided by this central body (akin to a methods article) rather than an individual article (e.g., used BRFSS Data Quality Reports from each year).

Survey name: Record survey name and acronym (used in survey materials, articles using the data, or communication with survey administrators or data users). For non-English surveys, record survey name, acronym as used in available English documentation.

Year: record year started & ended for multi-year surveys. For multi-site surveys (e.g., WMHS, GENACIS) record the earliest and latest years in which the survey was in the field in any nation.

Mode of Administration: PAPI = Paper and pencil interview, PAPQ = Paper and pencil self-completed questionnaire, CAPI = Computer assisted personal interview, CATI = Computer assisted telephone interview, CASI = Computer assisted self-interview, PATI = Paper and pencil telephone interview, PI = Personal interview of undetermined type, TI = Telephone interview of undetermined type. If an included article or other source notes that different modes of administration were used for childhood maltreatment content, compared to the remainder of the survey (e.g., in OHSUP), multiple modes were recorded. Similarly, record multiple modes if sampled respondents from remote regions had different administration of the survey instrument (e.g., GSS).

N: Record the unweighted number of participants in the representative sample. If nonrepresentative subsamples were collected through other means (e.g., MIDUS twin, sibling subsamples) do not include the subsamples N.

Ns represent unique samples except for NCS-2 (2001-03) and MIDUS-2 (2004-06) which were follow-up surveys of the samples used in NCS and MIDUS.

Response Rate: Where sources provided multiple response rates calculated using diverse formula, the method suggested by the Council of American Research Organizations (CASRO) was used.^[[2]](#footnote-2)^

Age: Record the age range of participants who completed the survey.

4. b. Tables 2-6 (common childhood maltreatment content)

For all five types of childhood maltreatment, these coding rules were followed:
Nonsexual victimization (including exposure to family violence) that was family-related, that occurred in childhood (before the 18^th^ birthday) was coded. Sexual victimization that occurred in childhood (before the 18^th^ birthday) was coded. The types (identities) of perpetrators to be described could be specified on a separate item or be defined as part of the question (e.g., “People in my family…”).

Precision in description and interpretation was always a goal. Example 1: the British National Survey of Psychiatric Morbidity (2000) included the item “sexual abuse” in a list of possible lifetime victimizations. Although “sexual abuse” was assumed in an accompanying editorial (Fullilove, 2009) to refer to childhood sexual abuse, the item and survey were not included in this review because the question asked of respondents did not specify “childhood” nor ask age at occurrence (Bebbington et al., 2004; 2009). Bebbington et al. 2004 p. 224 “the timing of [adverse life] events was insufficiently demarcated.” A later cycle of the British National Survey of Psychiatric Morbidity (APMS 2007) was included because participants were asked to indicate age at occurrence.

Example 2: The Behavior Risk Factor Surveillance System (BRFSS) – Texas – 2008 included four questions about unwanted sexual experiences that occurred any time in the respondent’s life. One of these allowed coding of the perpetrator as parent, parent’s partner, or equivalent. This item could not be included in this review because age at occurrence nor occurrence during childhood (versus adulthood) could be unequivocally determined. It is possible that a sexual assault by a parent or equivalent could have occurred past the age of 18 years.

To enhance precision, footnotes in Tables 2-6 were used to indicate situations where coded items differed slightly from column headers.

Some surveys included victimization items to be answered for lifetime experiences. As long as it would be possible for a researcher to isolate experiences occurring during childhood (e.g., question asks “how old were you when/the first time/the last time) this happened to you?”) then the survey item is included here.

An “item” is a question on a survey that assesses any aspect of childhood maltreatment, including the age at which it occurred, the perpetrator, or the harmfulness of the experience. Each bullet point in a list of possible experiences is counted as one item, if and only if a respondent can respond y/n or with a frequency to every item. Multiple concepts (e.g., violent acts) may be assessed within one item (e.g., “beat up,” “choked,” “burned/scalded”). The content of one item may thus be coded across multiple columns. “Stems” to questions to which no answer is made are not counted as items. Surveys on which respondents can describe multiple perpetrators may ask the same items for each perpetrator but the item number to be recorded reflects the number of unique items.

An item may include multiple concepts such that respondents’ endorsement of the item cannot be understood as indicating any one concept. For example, the NCS included the behaviours “beat up,” “choked,” “burned/scalded” on one item. These concepts are coded separately in our physical abuse table because our purpose is to highlight to researchers on what surveys what types of child maltreatment are assessed, and the nature of those assessments. The use in this review of multiple columns to describe many diverse child maltreatment behaviours does not imply that any particular survey could be used to look at a particular child maltreatment behaviour (e.g., “choked”) in isolation from other types of child maltreatment behaviours. It indicates only that a behaviour was included on a survey.

“Self-defined” = any of a variety of maltreatment experiences could be captured by the item, depending on respondents’ interpretations (“sexually attacked you” “other – not listed above – unwanted sexual acts”). One or more items may include specific behaviours, but there is also broad scope for respondents to interpret. Self-defined may mean that there are only one or two items which list no specific behaviours. Or also use this column when there are behaviours specified but also scope for participants’ interpretation (e.g., “or other sexually violent acts”).

Nine of the 47 surveys assessing sexual abuse included exclusively a self-definition item. GENACIS, SUSY, ASHR1, ASHR2, BRFSS CA 2010, USUMA 2005, 2007, 2008, BNAS

A self-definition item may be self-defined but also specify that noncontact forms of abuse are to be included.

“Other” = we do not have a category (column) for this specific concept. “Other’ is used only when a specific maltreatment behaviour that is not in the column headers is identified. The use of “other” was minimized by creating new columns as not previously coded maltreatment behaviours were found, over the course of the review.

There is more than one indication in a box when some items on the survey are assessed in one way and some in another (e.g., C, RD) for “specification of childhood.”

• = the survey assessed this characteristic on one or more items;

– = the survey did not assess this characteristic on any item;

<# = survey defines childhood as the years before this birthday.

P = perpetrator is defined within one or more items as parent or person who raised the respondent;

ID = respondents identified the perpetrator in terms of their relationship;

U = perpetrator is unspecified (e.g., “people in my family,” people in my home);

C = when respondent was growing up, during childhood or adolescence;

RD = precise age at occurrence of maltreating experience was recorded and thus researchers can define at what ages a person experienced maltreatment during “childhood” (e.g., before age 18, before age 16)

A= survey specifies that an adult in your home was the perpetrator;

Immediate harm: a question about help-seeking does not count unless it is exclusively immediate (e.g., required medical attention). The experience of maltreatment led immediately to injury or to medical visit. Do not code “did you ever go to a doctor” because this could involve therapy sought years later, but code “did you have to get medical attention” due to implication of immediacy.

Items in the Australian Study of Health and Relationships did not count even though “at a hospital” is one of the response options, because it is not necessarily immediate help seeking; could be counselling sought through a hospital program years later.

Note RE cross-national surveys:

Coding childhood maltreatment items included on cross-national surveys was complicated because survey content sometimes varied between nations: some nations did not include on their surveys items included on other nations’ versions of the survey.

- Regarding WMHS: Australia is not part of this cross-national sample (<http://www.hcp.med.harvard.edu/wmh/national_sample.php>) but a collaborative project there in 2007 called the Australian National Survey of Mental Health and Wellbeing used the CIDI and assessed family violence, physical abuse, and sexual abuse. Tables 2-6 were populated based on the NCS-R (the WMHS version used in the United States), because it was available <http://www.hcp.med.harvard.edu/ncs/replication.php>.
- GENACIS has a list of which incarnations of the surveys (i.e., in which countries) the childhood maltreatment item (sexual abuse) was included. However, contact with WMHS representative indicated that no such list exists for WMHS. Therefore a list of countries that included child maltreatment items on the WMHS was compiled based on captured articles.

All child maltreatment content had to be coded as one of the five types. In BRFSS Florida 2010 a question about “childhood abuse” was included on the survey but could not be coded here because it could not be categorized as any of the five types.

c. Table 2: Neglect

Difficulties in defining and measuring neglect have been previously noted. MacMillan et al. (2013) noted the challenges in measuring the occurrence of neglect retrospectively in large community-based surveys. Similarly, Dubowitz et al. (2005) noted the heterogeneity of types of neglect and labelled it an “inherently complex phenomenon” (p. 173).

Some items coded as “neglect” were embedded within modules assessing the quality of childhood relationships with parents (e.g., NCS, MIDUS, WMHS, USUMA 2006). For the NCS two of eight questions about respondents’ relationships with their mothers and father were coded as neglect questions given the two questions’ similarity to previously-used measures of neglect as a lack of a close and confiding relationship with any adult in childhood (Sareen et al. 2005).

Also note that Michal et al.’s (2009) article using the Quality of Recalled Parental Rearing Behavior (QRPB) and the survey on which it was based were included. The QRPB assesses rejection & punishment; emotional warmth & control; overprotecting. We took three items as indicative of emotional abuse, neglect, and physical abuse because they were a good fit with Michal et al. noted that their findings were consistent with those examining childhood maltreatment and because three QRPB’s items’ were similar to items captured by these three checklists. “You were comforted by your parents when you were sad?” is taken as the reverse of “lack of attention.” Similarly, being hit was an item included as physical abuse, and being punished severely for little reason was an item included as emotional abuse.

A respondent could say “yes” to a “close confiding relationship” question but still have been emotionally neglected by parents. But a “no” to an item indicating there was no close confiding relationship with any adult in the respondents’ childhood indicates emotional neglect. Despite the complexity of judging what counts as items indicating emotional neglect, they were included because such items might be useful to neglect researchers.

For NCS-2, Item read: Was the communication between you and mother/father excellent, good, fair or poor?” = not coded as an item indicating childhood neglect because poor communication may not equal emotional neglect. In contrast, an item from MIDUS read: “How much could you confide in mother/father about things that were bothering you?" and this was coded as “close, confiding relationship” which, reverse scored, can indicate childhood emotional neglect.

(R) = reverse coded item.

Because neglect is the absence of care & protection, and is sometimes assessed via negative answers to care and protection questions, identification of perpetrators is more complex than active forms of child maltreatment. If neglect items on a particular survey were exclusively reverse scored, no perpetrator codes were assigned.

d. Table 3: Emotional Abuse (EA)

Perceived hostility from parents and similar items were included as emotional abuse, as were threatened physical violence. “Threatened with a gun or other weapon” is an item used on multi-item measures of childhood maltreatment in seven surveys. It was included on surveys only with other physical abuse items. It was coded on our physical abuse checklist rather than as a type of emotional abuse. Despite the apparent inconsistency with other “threats” on the emotional abuse checklist, to have included “threatened with a gun or weapon” on the emotional abuse checklist would have added six surveys to the emotional abuse checklist based only on one item, and would seem to artificially inflate the number of surveys assessing emotional abuse.

e. Table 4: Exposure to Family Violence (EFV)

Violent/abusive acts witnessed and/or occurring in household. “Violence” here includes emotional abuse (e.g., insults) as well as physical violence, but not “conflict” if no abuse or violence is specified.

Self-defined item read like this: “family violence” “serious physical fights at home” “serious fights” “violence in the home” “parents physically assault each other”

Directly witnessed = “directly witnessed” is language in question or “see or hear” is language in question (e.g., CCHS)

“Beat up” is coded when the item reads “beaten,” “beat up” or “repeatedly hit.”

IPV = violence was between respondent’s parents or respondent’s parent and the parent’s intimate partner;

M = violence against respondent’s mother (or mother substitute);

A = survey specifies that “an adult” was the perpetrator or victim;

f. Table 5: Physical Abuse (PA)

Physical violence inflicted by a parent, guardian, or caregiver was included but violence inflicted by “anyone” or “an adult” was not included. Survey administrators for the NAS included an item as “childhood physical abuse.” We do not include the NAS item because we defined childhood maltreatment as within the home except for sexual abuse. Similarly we do not include the CCHS 2012 items on physical abuse, (“any adult” “in your school, in your neighbourhood, or in your family”) with no perpetrator item. In contrast, the same physical abuse items on GSS 2014 are included because the survey includes a perpetrator identity item. Also the (Canadian) National Population Health Survey 1994 is excluded due to lack of a perpetrator item and no specification of family in the item “…someone close to you”.

If “parent or other adult in your home” is specified in the question, count as PA.

“Physically attacked” is an example of a “self-defined” item (e.g., NESARC2);

“Beat up” is coded when the item reads “beaten,” “beat up” or “repeatedly hit.”

X = an item instructed survey participants *not* to respond in terms of spanking or hitting that was for the purpose of educating them or correcting their behaviour

A= other adult in the home.

g. Table 6: Sexual Abuse (SA)

We included any sexual abuse that was inflicted before age 18, not exclusively acts committed by a caregiver. We made this decision because the intent of the overall review was to take a broad perspective and to identify and evaluate childhood maltreatment measures used on population representative surveys. To restrict sexual abuse to acts committed by a caregiver would have made our criteria for sexual abuse in keeping with our criteria for other types of childhood maltreatment. However, the high frequency of occurrence and the harmfulness of sexual abuse that is extrafamilial is widely recognized. Thus, to restrict to caregiver-inflicted sexual abuse would have meant that many surveys’ assessments of health-relevant childhood maltreatment was excluded.

Frequency for sexual abuse: usually means the survey included an item that participants used to indicate how often something happened (i.e., rather than just y/n). If items assessed age at onset and offset of sexual contact, code as frequency. If participants could record multiple ages at which sexual assault happened, code as frequency.

Although ages of respondent and respondent’s partner at first intercourse might be used as an indicator of childhood sexual abuse by some researchers, any surveys that included such questions (e.g., SIS) also included more specific items about various sexual acts respondents might have experienced against their will during childhood. Therefore we didn’t code “age at first intercourse” questions here.

Any item assessing pregnancy resulting from rape is to be coded as an item assessing “harm.”

Noncontact means: Made to expose self, witness other exposing self, witness other masturbating, unwanted sex talk, watch sexual things (films, photos, internet content), approached sexually.

Receive oral/genital contact: Coded only if questionnaire item explicitly specified receptive contact. Note that NVAWS, NSHIS and RS put receptive and intrusive oral contact on same item.

A = survey specifies that “an adult” or someone 5+ years than respondent was the perpetrator of maltreatment.

5. Reliability/validity search and information extraction

1. Supplemental search

Data abstraction from included articles by two coders included information about each article’s description of the reliability and/or validity of the child maltreatment items used in that survey. Reviewing relevant sources cited by included articles was the initial step. Survey websites (where available) were also searched. Additional materials pertaining to reliability and validity of surveys’ child maltreatment assessments were obtained by other Internet searching (using key words from each survey title in combination with “reliability” and “validity”).

Summary of reliability/validity findings:

General Social Survey (GSS 2014) – see CEVQ and CEVQ-SF reliability testing, for three physical abuse items. Tanaka et al., 2012; Walsh et al., 2008

Ontario Health Survey, Mental Health Supplement (OHSUP) – Chartier et al. 2009) (N458) indicates that the PA measures are valid because they’re from the CTS, but they are modified, which means previous validity testing may not apply. They also indicate that good reliability and validity were found for childhood sexual abuse items drawn from the National population survey of Canada.

National Comorbidity Survey (NCS) – preliminary studies were done to increase respondent recall accuracy (360; 440). Authors have referred to the items drawn from the CTS as being valid, but they are modified so the reliability/validity testing of the CTS may not apply to them.

National Health and Social Life Survey (NHSLS) – steps taken to increase participants’ comfort with/accuracy of responding is suggested to makes the measure more accurate. This is not counted for purposes of this review to indicate reliability nor validity.

MIDUS – Authors have referred to the items drawn from the CTS as being valid, but they are modified so the reliability/validity testing of the CTS may not apply to them.

SAVI – pilot tested, Face validity. This is not counted for purposes of this review to indicate reliability nor validity.

WMHS – CIDI is valid for psychiatric diagnoses, but no mention of validity of trauma questions

USUMA 2006 –one article states that the scale used is reliable and valid.

USUMA 2010 – construct validity, internal consistency good for all measures except physical neglect.

APMS – in interviewer training interviews were repeated by a supervisor for quality control. This is not counted for purposes of this review to indicate reliability nor validity.

Korean GSS – Jeon et al. 2014 noted earlier work that established the reliability and validity of the childhood maltreatment measures.

**References**

Bebbington PE, Bhugra D, Brugha T, Singleton N, Farrell M, Jenkins R, et al. Psychosis, victimisation and childhood disadvantage: evidence from the second British National Survey of Psychiatric Morbidity. Brit J Psychiat. 2004; 185(3):220-226.

Bebbington P, Cooper C, Minot S, Brugha T, Jenkins R, Meltzer H, et al. Suicide attempts, gender, and sexual abuse: data from the 2000 British Psychiatric Morbidity Survey. Am J Psychiat 2009; 166:1135-1140.

Chartier, Mariette J., John R. Walker, and Barbara Naimark. "Health risk behaviors and mental health problems as mediators of the relationship between childhood abuse and adult health." American Journal of Public Health 99, no. 5 (2009): 847.

Dubowitz H, Newton RR, Litrownik AJ, Lewis T, Briggs EC, Thompson R, et al. Examination of a conceptual model of child neglect. Child Maltreat 2005; 10(2):173-189.

Fullilove M. Toxic sequelae of childhood sexual abuse. Am J Psychiat 2009; 166:1090-1092.

Jeon, Hong Jin, Christina Lee, Maurizio Fava, David Mischoulon, Eun-Jung Shim, Jung-Yoon Heo, Hong Choi, and Jae-Hyun Park. "Childhood trauma, parental death, and their co-occurrence in relation to current suicidality risk in adults: a nationwide community sample of korea." The Journal of nervous and mental disease 202, no. 12 (2014): 870-876.

MacMillan, H. L., Tanaka, M., Duku, E., Vaillancourt, T., & Boyle, M. H. (2013). Child physical and sexual abuse in a community sample of young adults: Results from the Ontario Child Health Study. Child abuse & neglect, 37(1), 14-21.

Michal M, Wiltink J, Subic-Wrana C, et al. (2009) Prevalence, correlates, and predictors of depersonalization experiences in the German general population. J Nerv Ment Dis 197: 499-506.

Sareen J, Fleisher W, Cox BJ, Hassard S, et al. (2005) Childhood adversity and perceived need for mental health care: findings from a Canadian community sample. J Nerv Ment Dis 193: 396.

Tanaka, M., Wekerle, C., Leung, E., Waechter, R., Gonzalez, A., Jamieson, E., & MacMillan, H. L. (2012). Preliminary evaluation of the Childhood Experiences of Violence Questionnaire short form. Journal of Interpersonal Violence, 27(2), 396-407.

Walsh, C. A., MacMillan, H. L., Trocmé, N., Jamieson, E., & Boyle, M. H. (2008). Measurement of victimization in adolescence: Development and validation of the Childhood Experiences of Violence Questionnaire. Child Abuse & Neglect, 32(11), 1037-1057.

1. The same inclusion criteria were applied to the survey search and the article search, except that surveys conducted in languages other than English were included if they were described in an article written in English and if materials were available in English or if communication with survey administrators in English was possible. These non-English surveys were included following contact with authors and/or survey administrators: Baromètre Santé [3 cycles], NEMESIS [2 cycles], Sex in Sweden [1 cycle], German surveys [5 surveys], Brazilian National Alcohol Survey [2 cycles], Korean General Social Survey [1 cycle]. [↑](#footnote-ref-1)
2. Explanation of calculation of MIDUS 1 & MIDUS 2 response rates.

   To be consistent with how we report response rates in Table 1, we want to report:

   1) For only the nationally representative part of the sample (not city oversample, nor twin nor sibling subsamples)

   2) We want the response rate, out of all people approached to participate, to show how many of those approached ended up answering the childhood maltreatment questions. The child maltreatment questions were in the SAQ (mailed questionnaire) for MDUS 1 & 2. For MIDUS 2 we want the percentage of respondents to the original survey who answered the SAQ in MIDUS2.

   3) We need to calculate the total number of people approached to participate in RDD sample.

   a. MIDUS 1 = 3487/0.7 = 4981. Out of them, 3034 answered SAQ = 61%

   b. MIDUS 2 = 2257/0.71 (rr adjusted for mortality) = 3179. Of these 3179 MIDUS 1 main RDD sample people approached, 1805 people completed the SAQ. 1805/3179 = 57% [↑](#footnote-ref-2)
